# Supplementary material for: A new method for detecting mixed Mycobacterium tuberculosis infection and reconstructing constituent strains provides insights into transmission
Source: Genome Med. 2025 Jan 27;17:8. doi: 10.1186/s13073-025-01430-y (PMC11771024; doi:10.1186/s13073-025-01430-y)
Supplement: Supplementary file 1 — Additional file 1. Contains supplementary figures S1 – S5 of additional results. [file 13073_2025_1430_MOESM1_ESM.docx]

**A new method for detecting mixed *Mycobacterium tuberculosis* infection and reconstructing constituent strains provides insights into transmission – Supplementary Figures**

Benjamin Sobkowiak^1,2*^, Patrick Cudahy^3^, Melanie H. Chitwood^1^, Taane G. Clark^4,5^, Caroline Colijn^6^, Louis Grandjean^2^, Katharine S. Walter^7^, Valeriu Crudu^8^, Ted Cohen^1^

1. Department of Epidemiology of Microbial Disease, Yale School of Public Health, 60 College Street, New Haven, CT, USA.

2. Department of Infection, Immunity and Inflammation, Institute of Child Health, University College London, London, UK.

3. Division of Infectious Diseases, Department of Internal Medicine, Yale School of Medicine, New Haven, CT, USA.

4. ﻿Faculty of Infectious and Tropical Diseases, London School of Hygiene and Tropical Medicine, UK.

5. Faculty of Epidemiology and Public Health, London School of Hygiene and Tropical Medicine, UK.

6. Department of Mathematics, Simon Fraser University, 8888 University Drive West, Burnaby, BC, Canada.

7. Division of Epidemiology, University of Utah, Salt Lake City, UT, USA.

8. Phthisiopneumology Institute, Strada Constantin Vârnav 13, Chisinau, Republic of Moldova.

*Corresponding author: [bs2259@yale.edu](mailto:bs2259@yale.edu).


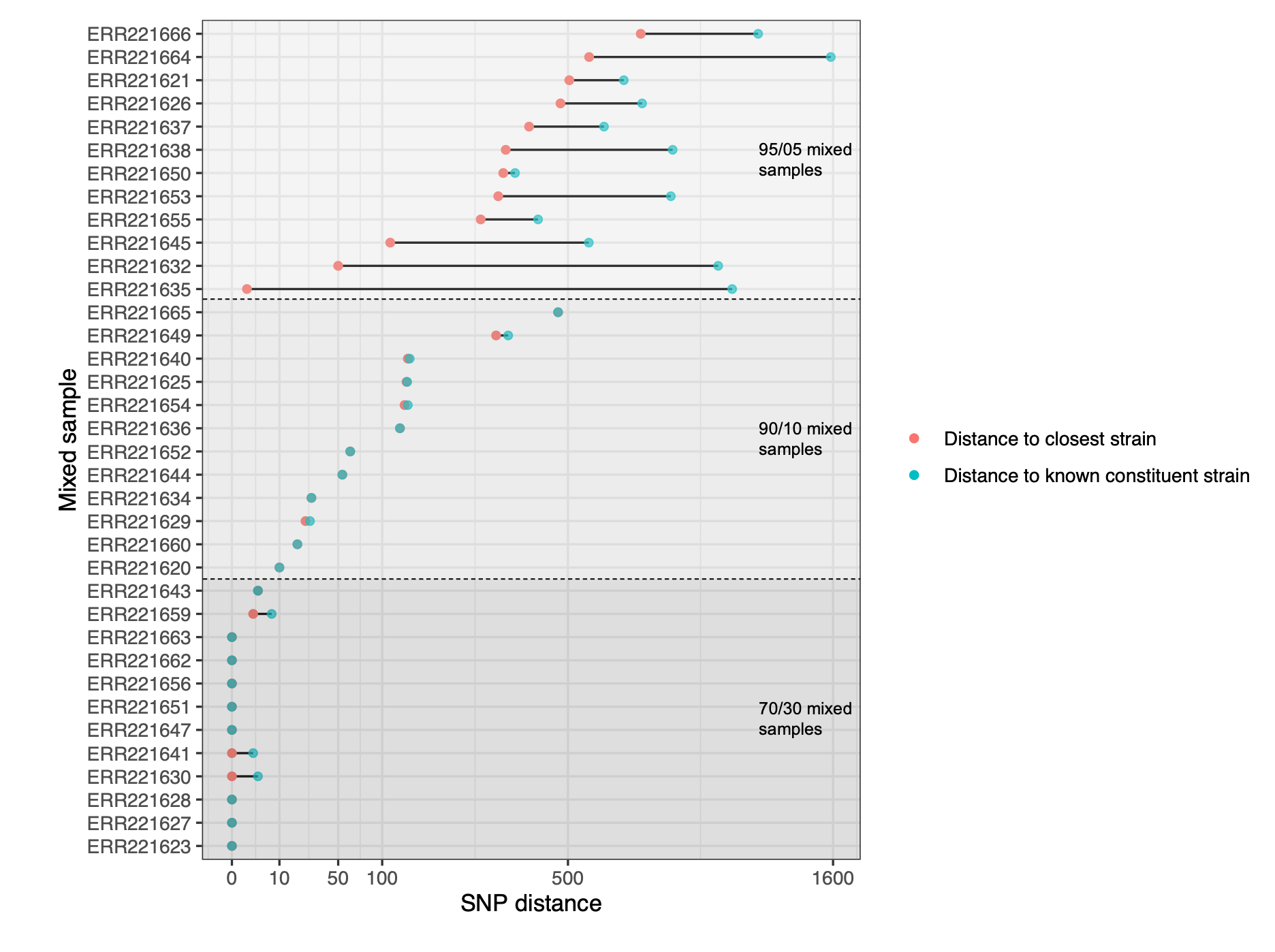


**Fig. S1**. The SNP distance between the closest identified strain sequence in the reference database of ‘pure’ isolates in the Karonga dataset and the known constituent minor strain sequence in each *in vitro* mixed sample.

**
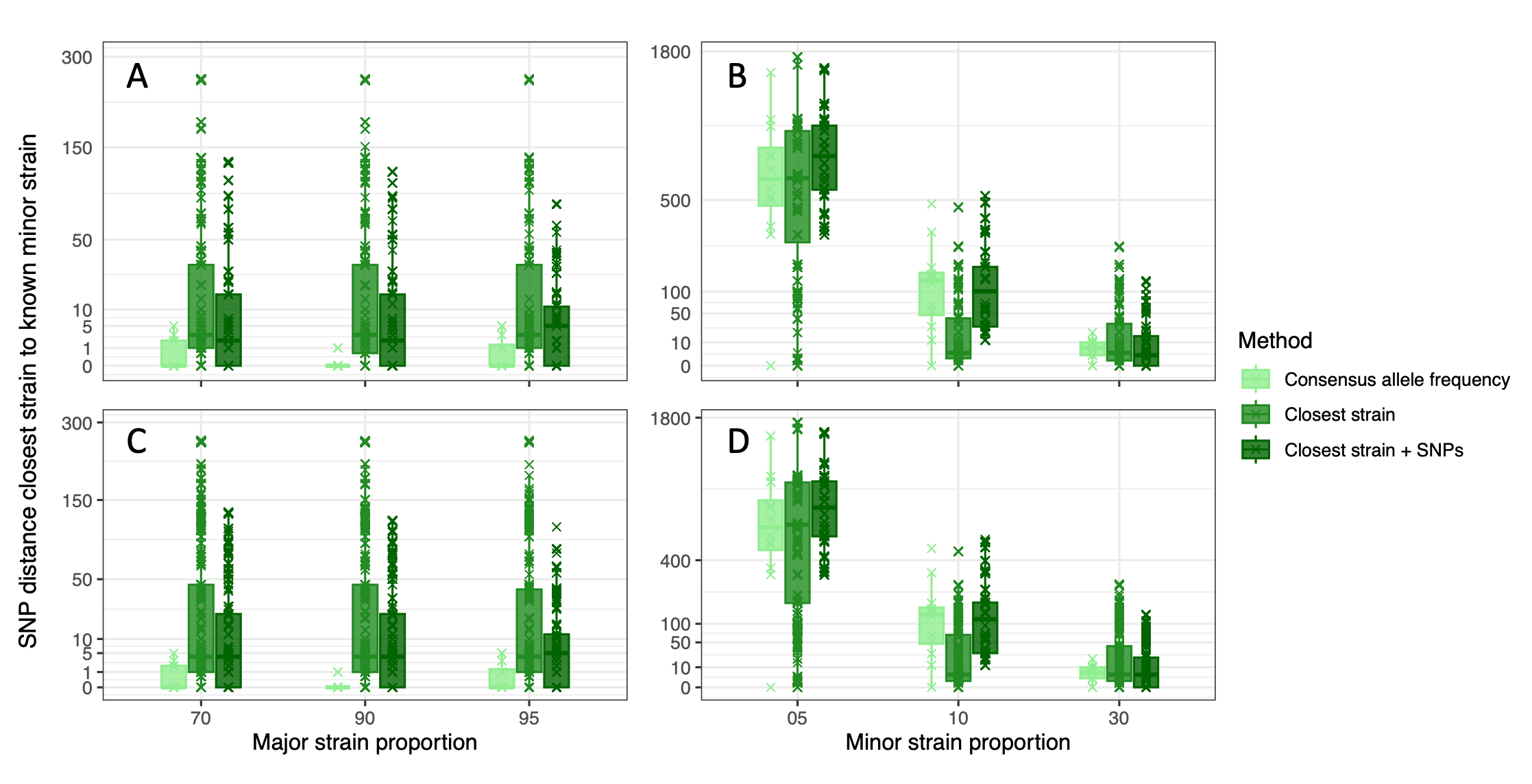
**

**Fig. S2**. Boxplots showing the SNP distance between the predicted and known constituent strains of *in vitro* mixed samples using the three tested approaches when downsampling the reference dataset of ‘pure’ strains by 50% and 75%, with 100 replicates per downsample strategy. Plots **A** and **B** show the major and minor strain estimates with the 50% downsampled ‘pure’ dataset and plots **C** and **D** show the major and minor strain estimates with the 75% downsampled ‘pure’ dataset. Known constituent strains were not included in the ‘pure’ strain datasets. Boxes are colored by the different methods used to predict the constituent strains. Note that the Y axes have been transformed by the square root for visualization.


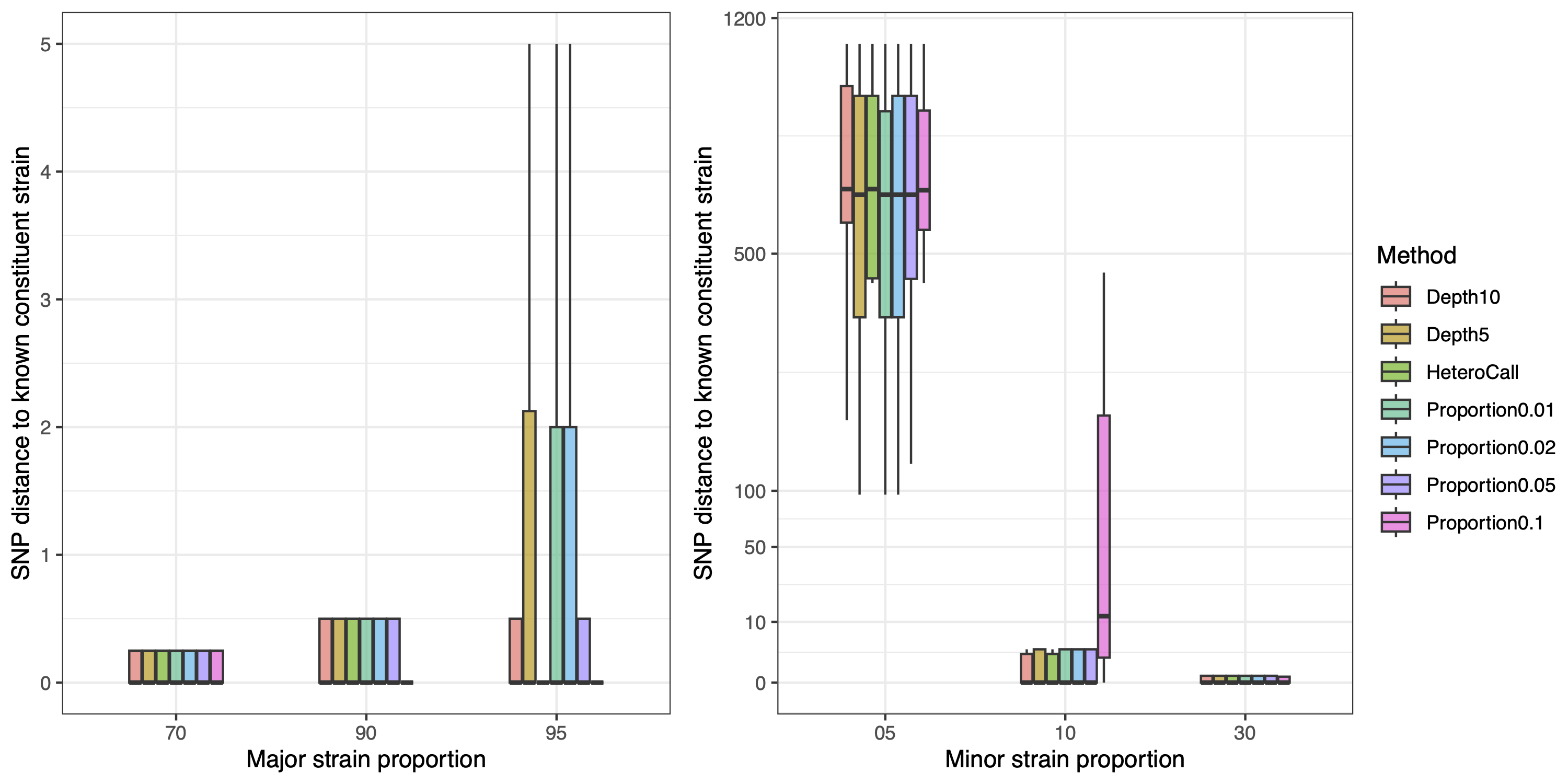


**Fig. S3**. The distance between the closest identified strain and the known constituent strain using seven different approaches to characterizing hSNPs for the major strain sequence (A) and minor strain sequence (B) of the *in vitro* mixed samples.

**Fig. S4**. A maximum likelihood phylogenetic tree of the real-world Mtb samples from Moldova including the reconstructed constituent strains of predicted mixed infections. Terminal branches colored by the infection status of the sample at the tip, the non-mixed ‘pure’ strains in yellow, the major constituent strains of mixed infections in blue, and the minor constituent strains of mixed infections in red.

**Fig. S5**. An example section of a binary alignment (BAM) file, visualized using Tablet ^37^, from a Moldova *Mtb* isolates (TB-222-6565-19_S218) that was not classified as mixed but harbored a high number of hSNPs. Variants found in aligned reads against the H37Rv reference sequences are highlighted, showing multiple hSNPs close in the genome and coverage almost double the average for surrounding regions, shown within the red circle.
